# Endocannabinergic modulation of central serotonergic activity in healthy human volunteers

**DOI:** 10.1186/s12991-023-00437-2

**Published:** 2023-03-17

**Authors:** Barbara Emons, Larissa Arning, Vera-Estelle Makulla, Maria-Theresia Suchy, Dimitrios Tsikas, Thomas Lücke, Jörg T. Epplen, Georg Juckel, Patrik Roser

**Affiliations:** 1 Department of Psychiatry, Psychotherapy and Preventive Medicine, LWL University Hospital, Ruhr-University Bochum, Alexandrinenstr. 1-3, 44791 Bochum, Germany; 2 Department of Human Genetics, Ruhr-University Bochum, Bochum, Germany; 3 Institute of Clinical Pharmacology, Hannover Medical School, Hanover, Germany; 4 Department of Neuropediatrics, St. Josef-Hospital, Ruhr-University Bochum, Bochum, Germany

**Keywords:** Serotonergic system, LDAEP, *CNR1* gene, *FAAH* gene, Endocannabinoids (2AG, AEA), Depression

## Abstract

**Background:**

The serotonergic and the endocannabinoid system are involved in the etiology of depression. Depressive patients exhibit low serotonergic activity and decreased level of the endocannabinoids anandamide (AEA) and 2-arachidonylglycerol (2AG). Since the cannabinoid (CB) 1 receptor is activated by endogenous ligands such as AEA and 2AG, whose concentration are controlled by the fatty acid amide hydrolase (FAAH) and monoacylglycerol lipase, respectively, we investigated the effects on serotonergic utilization. In this study, we investigated the impact of the rs1049353 single-nucleotide polymorphism (SNP) of the cannabinoid receptor 1 (*CNR1*) gene, which codes the endocannabinoid CB1 receptor, and the rs324420 SNP of the *FAAH* gene on the serotonergic and endocannabinoid system in 59 healthy volunteers.

**Methods:**

Serotonergic activity was measured by loudness dependence of auditory-evoked potentials (LDAEP). Plasma concentrations of AEA, 2AG and its inactive isomer 1AG were determined by mass spectrometry. Genotyping of two SNPs (*rs1049353, rs344420*) was conducted by polymerase chain reaction (PCR) and differential enzymatic analysis with the PCR restriction fragment length polymorphism method.

**Results:**

Genotype distributions by serotonergic activity or endocannabinoid concentration showed no differences. However, after detailed consideration of the *CNR1*-A-allele-carriers, a reduced AEA (A-allele-carrier *M* = *0.66*, SD = *0.24*; GG genotype *M* = *0.72*, SD = *0.24*) and 2AG (A-allele-carriers *M* = *0.70*, SD = *0.33*; GG genotype M = 1.03, SD = *0.83*) plasma concentration and an association between the serotonergic activity and the concentrations of AEA and 2AG has been observed.

**Conclusions:**

Our results suggest that carriers of the CNR1-A allele may be more susceptible to developing depression.

## Introduction

Major depression is among the most common and in terms of its severity the most underestimated disease. The fact, that the disease impacts the well-being and the quality of life, thus causing a high psychological strain [30]. Major depression is characterized by mental dejectedness as lead symptom and shows cyclic courses. Further symptoms can be detected in the emotional, motivating, cognitive and neuro-vegetative area and in the psychomotor activity [2]. The manifestation of the disease varies while the symptomatology shows a common characteristic [42]. Depressive diseases are heterogeneous illnesses, which can be traced back on the interaction of multiple genes with environment factors such as stress [10, 35, 31].

Disorders of the serontonergic system are being discussed as pathogenetic factors for the emergence and characteristic of psychiatric diseases such as depression [48]. In the pharmacological treatment of depression, the serotonergic system has proven to be an effective point of action for therapy [9]. The monoamine hypothesis of depression is the most popular theory, it states that a reduced monoaminerge transmission within the central nervous system is the reason for depression [52]. The successful application of serotonin and noradrenalin-based antidepressants like selective serotonin reuptake inhibitors (SSRIs) confirms this hypothesis [51]. The defective regulations of the serotonin balance during a depression phase are found in different parts of the serotonergic system [38, 53].

The endocannabinoid system plays an important role in the modulation of the serotonergic system [29]. There are two cannabinoid (CB) receptors (CB1 and CB2) in the endocannabinoid system, whose natural ligands and specific enzymes are responsible for their biosynthesis and metabolic inactivation. It has been shown that the endocannabinoid system is involved in the pathophysiology of depression [25, 54, 57]. The CB1-receptor is a G-protein-coupled receptor, which is expressed in the central nervous system [19] and plays a role in the serotonergic neurotransmission [4, 20]. Five endocannabinoids exists, whereof Anandamid (AEA) and 2-Arachidonoylglycerol (2AG) are the most potent substances. Endocannabinoids can be considered as neuromodulators at the CB1 receptor [14].

Several genetic studies show that there is an interaction between the CB1 receptor gene (*CNR1*), the serotonin receptor gene (*SERT*) and the anxiety disorder [37]. Several experimental examinations in humans reveal a significant association between the functional (AAT)n-polymorphism within the *CNR1* gene and depression by patients with Parkinson’s disease [6]. It is, however, assumed that a changed *CNR1* expression plays an important role for the pathogenesis of depression [57]. A lower CB1 receptor density has been found in the cortex of patients with major depression [36]. The *CNR 1* rs1049353 A allele exhibits an increased risk for developing a depression particularly in haplotypic combination [34]. The effect of variation within the *CNR1* gene for depression have been shown [43]. Furthermore, the CNR1 gene is negatively regulated by glucocorticoids. Hypercortisolemia is present in depressed patients, so this supports the hypothesis that there is decreased activity of the endocannabinoid system in these patients [39]. The CNR1 polymorphism studied, rs 1049353, could influence CNR1 receptor functions by affecting translation or mRNA stability [11].

The CB-1 receptor is activated by endogenous ligands such as AEA and 2AG. The hydrolytic enzymes Fatty Acid Amide Hydrolase (FAAH) and monoacylglycerol lipase (MAGL) are responsible for the metabolism of AEA and 2AG [12, 16]. In animal studies, CB1 receptor antagonist, endogenous cannabinoid reuptake inhibitor or inhibitor of FAAH enzymes generate an antidepressive effect [1, 20, 25]. The direct activation of central CB1 shows in animal models of depression (e.g. forced swim test, tail suspension test) a significant antidepressive effect [5, 20, 25, 56]. By analogy, depressive patients show a reduced 2AG-concentration in serum, whereby the concentration is negatively correlated with the duration of depression [28]. Single nucleotide polymorphism (SNP) in the rs324420*FAAH* genes lead to be relevant and have an impact on brain biochemistry, neurocircuitry, behavior and symptoms [15]. Moreover, this polymorphism reduces FAAH activity, resulting in increased anandamide serum concentrations [55].

Loudness dependence of auditory evoked potentials (LDAEP) is an established measure for the central serotonin-system activity [24, 33, 60]. A low LDAEP is implied by a high serotonergic neurotransmission [32]. A significant correlation in depressive patients is shown between the increased stimulus intensity of LDEAP measured with low serotonergic function and successful response on SSRIs [23, 32, 47]. Alterations in serotonin neurotransmission may increase the risk of psychiatric disorders, so “Loudness dependence of auditory evoked potentials” (LDAEP)—a non-invasive method may be applied to measure its activity. Preclinical and animal studies have shown that LDAEP is an indicator for serotonergic neurotransmission/activity in the brain [24, 33, 49, 60].

The aim of the present study was to investigate possible associations between the endogenous cannabinoid system and the central serotonergic activity in healthy volunteers. Central serotonergic transmission is determined indirectly by the loudness dependence of auditory evoked potentials. For this, we determine the distribution and effect of SNPs within the CNR1 gene and the FAAH gene and endocannabinoids concentration (AEA, 2AG) and the serotonergic activity.

Here, we hypothesize that *CNR 1* rs1049353 A carriers and in the presence of SNPs in rs324420*FAAH* genes a reduced AEA and 2AG concentration in plasma is present. In addition, we suspect reduced serotonergic activity in the presence of these.

## Methods

### Study population

The sample contained 59 healthy probands (31 females and 28 males, average age 28.47 ± 7.62 years). The test persons were right handed, normal hearing and non-smokers. Neither at the time of the survey nor in their health history they suffered from psychiatric diseases or acute, severe and unstable somatic illnesses. In their past, there was no substance abuse and no regular use of medicines. No psychiatric diseases of first degree relatives existed and there was also no low intelligence or suicidality. The investigation of psychometry was done by the following tests: Multiple Choice Word Test (MWT-B), Hamilton Depression Rating Scale (HAMD-21), Beck Depression Inventory (BDI), State-Trait Anxiety Inventory (STAI) und Brief Psychiatric Rating Scale (BPRS). Thereby, no psychiatric disorders were emerged. The mean score and the ranges of the psychometry are listed in the following: MWT-B 114.9 (90–129), HAMD-21 0.6 (0–4), BDI 1.54 (0–10), STAI 29.8 (20–58) and BPRS 18.1 (18–21).

### LDAEP measurement

To determine the central serotonin-system activity, the Loudness dependence of auditory evoked potentials (LDAEP) method was executed in a noise reduced and electrically shielded room adjacent to the measurement equipment. This was done via the subject´s eyes open using the Brain Vision BrainAmp ® MR method (Brain Products GmbH, Munich, Germany). The test persons sat in a slightly lying position on a chair with a headrest.

For the EEG acquisition, 32 non-polarized silver–silver chloride electrodes were attached to a suitable EEG cap (Easy Cap®) according to the 10/20 system (impedance ≤ 10 kΩ). The acoustic stimulation contained five stimulations with an inter-stimulus interval randomized between 1800 and 2200 ms. Tone bursts of 1000 Hz (Hertz) with a duration of 40 ms (milliseconds) (rise-/fade-time 10 ms) were presented to the test persons in 5 intensities (60, 70. 80, 90, 100 dB SPL) via headphones. The stimuli were created with E-Prime Software (Presentation 11.3® Neurobehavioral Systems Inc. Albany, CA, USA). The eye movement is captured by an electrode, which is placed 1 cm below the outside left corner of the eye. The impedance was kept below of 5 kΩ (kilo ohms). The EEG was recorded with a sampling rate of 256 Hz and an analogue bandpass filter (0, 16–70 Hz). At least 40 artifact-free trials per intensity were included in the further analysis (Brain Products GmbH, Munich, Germany). A semi-automatic measurement at the Cz (central zero) electrode was carried out at peaks of N1 (60–125 ms) and P2 (110–210 ms). The N1/P2 (negativ/positiv) amplitude was defined as the difference in the peak amplitude between N1 and N2. The calculation of LDAEP was executed by means of the least square linear regression slope, while the stimulus intensity was considered as the independent variable and the N1/P2 amplitude was considered as the dependent variable.

### Genotyping SNPs

DNA of the participants was extracted from EDTA (ethylendiaminotetraacetat)-anticoagulated peripheral blood by using a QIAamp DNA mini Kit (Qiagen GmbH, Hilden, Germany) under the protocol of Qiagen. SNPs were chosen for genotyping by selecting SNPs from the literature (*CNR1* rs1049353, *FAAH* rs324420). Genotyping/allel determination was conducted by polymerase chain reaction (PCR) and differential enzymatic analysis with the PCR restriction fragment length polymorphism method. Oligonucleotides were designed using Primer Express 2.0 Software (Applied Biosystems).

### Endocannabinoid measurements

Endocannabinoid measurements were performed at the Institute of Clinical Pharmacology, Hannover Medical School, Germany. Measurements were carried out on an ultra-performance liquid chromatograph model ACQUITY coupled with a tandem mass spectrometer (UPLC-MS/MS) model XEVO TQ MS (Waters, Milford, MA, USA) as reported elsewhere [61, 62]. We determined simultaneously the plasma concentration of AEA, 2AG and 1AG. Venous blood was collected early in the morning from subjects, who has fasted (not had anything to eat or drink) about 8 h. Venous blood samples were taken and drawn into EDTA-containing tubes (BD Vacutainer, Franklin Lakes, NY, USA) and centrifuged at 3500 rpm (rounds per minute). The time interval between blood sampling and centrifugation was kept below 10 min. Plasma aliquots were stored at − 80 °C until assay. The stored samples were thawed on ice and 300-µL aliquots thereof were spiked with a mixture of deuterated (d) analogs in ethanol, i.e., d_4_-AEA and d_5_-2AG, to reach a final concentration of 2.5 nM each. d_4_-AEA and d_5_-2AG, served as internal standards for AEA and 2AG, respectively. Then, plasma samples were incubated for 15 min on ice. Extraction was conducted by adding 1 mL toluene to each sample and by shaking twice in a Precellys®24 Dual homogenizer at 5000 rpm for 20 s with interruption of 5 s, so that the warming of the samples was prevented. The phase separation was carried out by centrifugation (4655×*g*, 4 °C, 5 min). The upper organic phase was transferred into a 1.5-mL glass vial. After solvent evaporation under a nitrogen stream, the residue was dried at room temperature (25 °C) under nitrogen. The residues were reconstituted in 40 µL of water–methanol (1:3, v/v) and mixed by vortexing for 10 s, and 10-µL aliquots were injected. Quantitative analyses were performed in positive electrospray ionization and selected–reaction monitoring modes as described previously [62].

### Statistical analysis

Pearson correlation analysis was used to determine the correlation of the whole sample and the eCS (endocannabinoids) concentration and the LDAEP value. For genotypes and alleles specific association with eCS concentration one-way ANOVA and t-Tests was conducted. SPSS Ver.21.0 for Windows (SPSS Inc.) was used for all statistical analysis. Data are reported as mean ± standard deviation (SD). *p* values less than 0.05 were considered statistically significant.

## Results

Healthy volunteers did not differ in age and an equal distribution existed for gender. The mean of the years of education was 16.56 ± 2.50. The mean score of the alcohol use was 2.6 drinks per week in a range of 0–15. Genotype and alleles distribution are displayed in Table 1. No psychiatric disorders were recorded. The central serotonergic activity, measured by LDAEP had a mean value of 0.252 ± 0.15. The consideration of the alleles distribution in matters of LDEAP showed, that the healthy volunteers with homozygous G (guanine) alleles of the rs 1049353*CNR1* gene in mean was a trend towards lower LDEAP score than the A (adenosine) allele carriers (0.233 ± 0.121 vs. 0.277 ± 0.169, *p* = 0.173). In case of the alleles allocation for the rs 324420*FAAH* gene, the homozygous C (cytosine) allele carriers displayed in mean a nearly identical LDAEP score than the A-allele-carriers (0.255 ± 0.161 vs. 0.243 ± 0.114) (Table 1).

**Table 1:** Genotype and allele distribution of the rs 1049353*CNR1* and rs 324420*FAAH* variations in healthy volunteers and mean and standard deviation of LDAEP score and concentration of the endocannabinoids (AEA, 2AG and totalAG) for the genotype and allele distribution

	Genotype			Group of genotype	
	GG	GA	AA	GG	GA + AA
*CNR1 (rs1049353)*					
*N* (%)	27 (40.5)	30 (54.8)	2 (4.7)	27 (40.5)	32 (59.5)
LDAEP (Mean, SD)	0.223 ± 0.12	0.272 ± 0.17	0.347 ± 0.93	0.223 ± 0.12	0.277 ± 0.17
AEA (nmol/L) (Mean, SD)	0.71 ± 0.23	0.65 ± 0.20	0.51 ± 0.08	0.71 ± 0.23	0.64 ± 0.24
2AG (nmol/L) (Mean, SD)	1.02 ± 0.82	0.66 ± 0.24	0.78 ± 0.16	1.02 ± 0.82	0.66 ± 0.24
TotalAG (nmol/L) (Mean, SD)	2.88 ± 3.02	1.50 ± 0.46	1.94 ± 0.59	2.88 ± 3.02	1.53 ± 0.47

	CC	CA	AA	CC	CA + AA
FAAH (rs324420*)*					
*N* (%)	44 (75.5)	13 (21.2)	2 (3.3)	44 (75.5)	15 (25)
LDAEP (Mean, SD)	0.255 ± 0.16	0.243 ± 0.12	0.247 ± 0.09	0.255 ± 0.16	0.243 ± 0.11
AEA (nmol/L) (Mean, SD)	0.64 ± 0.21	0.74 ± 0.17	0.90 ± 0.55	0.64 ± 0.21	0.76 ± 0.22
2AG (nmol/L) (Mean, SD)	0.76 ± 0.46	1.08 ± 0.90	0.42 ± 0.26	0.76 ± 0.46	0.99 ± 0.87
TotalAG (nmol/L) (Mean, SD)	2.01 ± 1.62	2.67 ± 3.49	1.40 ± 0.62	2.01 ± 1.62	2.50 ± 3.27

The plasma concentration was 0.67 ± 0.22 nmol/L for AEA, 0.82 ± 0.59 nmol/L for 2AG, and 2.13 ± 2.14 nmol/L for total AG, i.e., 2AG + 1AG (Table 1). Looking closer into the data of alleles distribution of the endocannabinoid concentration revealed that homozygous C allele carriers for the rs 324420*FAAH* gene displayed lower concentrations of the analysed endocannabinoids than the A-allele-carriers (AEA: 0.64 ± 0.21 nmol/L vs. 0.76 ± 0.22 nmol/L, *p* = 0.076; 2AG: 0.76 ± 0.46 nmol/L vs. 2AG: 0.99 ± 0.87 nmol/L, *p* = 0.199; total AG 2.01 ± 1.62 nmol/L vs. 2.50 ± 3.27 nmol/L, *p* = 0.452) (Table 1). There were no statistical differences detected when comparing genotype and allele frequencies related to the mean score of plasma endocannabinoid concentrations.

Group comparison of the LDAEP scores to evaluate the effects of the genotypes and alleles distribution of the focused genes the rs 1049353*CNR1* und rs 324420*FAAH* showed no statistical significances.

**Fig. 1:**
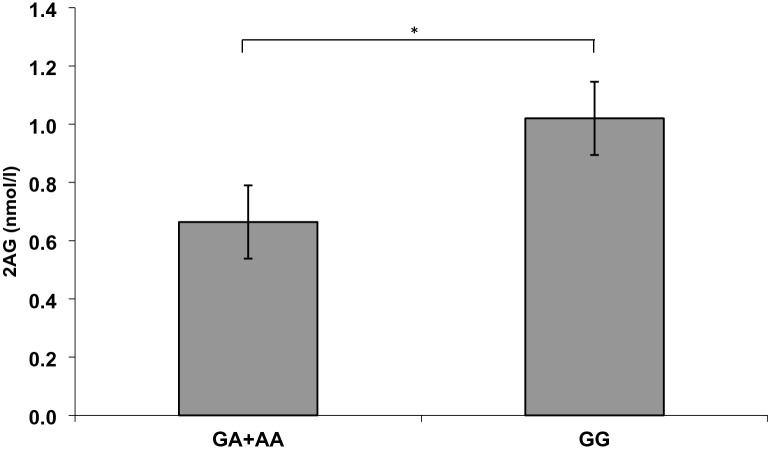
Plasma level comparison of the endocannabinoid 2AG between the A-allele carriers and the GG carriers of the rs 1049353*CNR1* variation (*p < 0.005, significant one-way ANOVA)

**Fig. 2:**
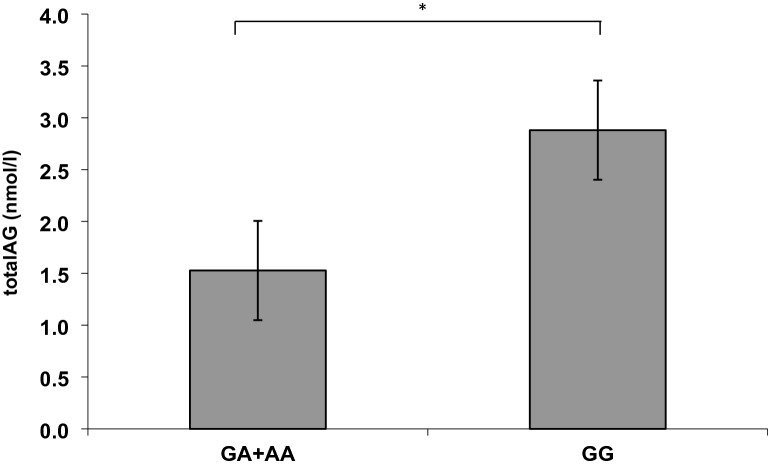
Plasma level comparison of the endocannabinoid total AG between the A-allele-carriers and the GG carriers of the rs 1049353*CNR1* variation. (*p < 0.005, significant one-way ANOVA)

Group comparison by *t* test of homozygous GG carriers and A carrier of the rs 1049353*CNR1* gene revealed a significant association of the *CNR1* and the 2-AG concentration (*t* = 2.352, *df* = 56, *p* = 0.022; Fig. 1), as well as of *CNR1* and total AG concentration (*t* = 2.500, *df* = 56, *p* = 0.015, Fig. 2) evident.

Correlation analysis of the whole population were calculated by Pearson, they detected no significant correlation between the LDAEP value and the concentration of the reviewed endocannabinoids (AEA, 2AG and total AG).

The *t* test of LDAEP scores to evaluate the effects of the genotypes and alleles distribution of the focused genes rs 1049353*CNR1* und rs 324420*FAAH* show no significances as well.

There was a tendency toward a negative for the subgroup of A-allele-carrier of rs 1049353*CNR1* gene in reference to the LDAEP value and the endocannabinoid AEA (*r* = − 0.185, *p* = 0.156). But the effect is not significant. In addition, for this subgroup, there was a trend toward negative correlation in terms of LDEAP values and the concentration of the endocannabinoid 2AG (*r* = − 0.234, *p* = 0.099).

## Discussion

The interaction between components of the endocannabinoid system and central serotonergic transmission in healthy volunteers was the basis of our studies, which examined, peripheral serum concentrations of the endocannabinoids 2-AG and anandamide and a single-nucleotide polymorphism of each of the CNR1 gene (rs1049353) and the FAAH gene (rs324420) and, LDAEP as an indirect measure of central serotonergic transmission.

The genetic aspects of polymorphism of central receptors or enzymes of the endocannabinoid system were of particular importance for the understanding of the processes. Delineating the underlying mechanisms improves our knowledge of depression and may contribute to better treat this disease. The present analysis investigating rs 1049353*CNR1* and rs 324420*FAAH* gene SNP variants in healthy volunteers. In about half of the healthy subjects, a homozygous G allele was present in the rs 1049353*CNR1* SNP studied. Whereas Monteleone et al. [44] displayed a three-quarter share in homozygous GG genotype of CNR1 gene in healthy controls. The difference of the distribution could be caused by the sample size. On the other hand, FAAH genotype distribution was nearly the same in both studies.

Evidence for the role of endocannabinoid signalling in the regulation of the serotonin system is suggested by behavioural studies showing a high level of functional overlap between the serotonin and the endocannabinoid system [40, 41, 46, 59].

Looking at the genotypes and alleles distribution of the SNPS rs 324420*FAAH* genes and rs 1049353*CNR1* in healthy volunteers of our study, no statistical differences were detected with respect to serotonergic activity. Nevertheless, Dlugos et al. [17] showed a genetic variation of rs 324420*FAAH* is associated with specific mood responses, whereas clinical studies displayed that genetic variability of the CNR1 gene predicts an effect to etiology of major depression and clinical response [43]. Different results between our and the groups are likely to be due to differences in the study populations, for instance, patients versus healthy volunteers in our study.

With regard to the genotypes distribution of rs 1049353*CNR1* and rs 324420*FAAH* genes in our study and the focus on the plasma concentration of AEA, 2AG and total AG, there were also no differences displayed in healthy volunteers. This also applies to the allele distribution of the *FAAH* gene. Whereas Chiang et al. [11] have shown that polymorphism in rs 324420*FAAH* determine in approximately half of the enzymatic activity of FAAH in humans, so that an increased activation of AEA and 2AG is triggered. As a consequence, the concentration of the investigated endocannabinoids had to be reduced. These results were also replicated in further investigations [15, 55]. In our study, we did not find higher AEA plasma concentrations suggesting that FAAH activity is not diminished in the healthy volunteers of our study. Following this line of investigation in animal models, a reduction of FAAH activity results in the increase level of endogenous cannabinoids AEA and 2AG, so an inhibition of the FAAH enzyme has antidepressant effect [20]. The CNR 1-A-allele exhibits an increased risk for developing a depression particularly in haplotypic combination [34]. A closer analysis of the allele distribution of the CNR1 gene, particularly the A-allele-carriers and the homozygous G-allele-carriers distribution, appears to show an association of the haplotype and the concentrations of the endocannabinoids AEA and 2AG. The measurement of the endocannabinoid concentrations in A-allele-carriers showed a lower concentration than in the homozygous G-allele-carriers. This is evident in various studies. Umathe et al. [56] showed an interaction between endocannabinoids (AEA, 2AG) and the serotonergic system in the regulation of depressive and compulsive behaviour. Reduced AEA and 2AG concentrations are determined in patients with major depression [26–28]. This means that a modification of the central endocannabinoid system caused a variation in major depression. Data of animal studies had already shown that regulation of either CB1 receptor antagonist, endogenous cannabinoid reuptake inhibitor or inhibitor of FAAH enzymes have generated an antidepressive effect [1, 20], [25]. The direct activation of central CB1 receptors with exogenous applied synthetic CB1-receptor agonists (e.g. HU210, WIN55, 212-2), by indirect activation of central CB1-receptor with selective FAAH inhibitors (e.g. URB597) or inhibitors of the membrane anandamid-transporter-receptor (e.g. AM404) showed in animal models of depression (e.g. forced swim test, tail suspension test) a significant antidepressive effect [20, 56, 25]. By administration of selective CB1-receptor antagonists (e.g. AM251, SR141716) nearby a CB1-receptor-mediated mechanism, a blocking of the effect is shown. By analogy, depressive patients show a reduced 2AG concentration in serum, whereby the concentration is negatively correlated with the duration of depression [28].

Further analysis of the *FAAH*-A-allele-carrier in terms of the dependency of the serotonergic activity and the plasma concentration of the investigated endocannabinoids indicated no interactions. Indeed, the same consideration of the subgroup of *CNR1*-A-allel-carriers showed specific coherencies. In this sense, it has been indicated that lower concentrations of the endocannabinoid AEA and 2AG came along with lower LDAEP score. These results point towards an influence of the *CNR1* genetic variation on the endocannabinoid concentrations and the serotonergic activity. Considering that *CNR1*-A-allele carriers have an increased risk to predict a major depression these results are accompanied with the findings of other studies that endocannabinoid concentrations were decreased in major depression [26–28].

Our findings were reinforced by the results in clinical studies. These studies showed an interaction between the endocannabinoid system and depression. The THC-analogue Dronabinol® showed antidepressive effects [8], while the selective CB1-receptor-antagonist SR141716 tended to result in depressive symptoms [13, 45]. The functional interaction of the serotonergic and endocannabinoid system in brain plays a potential role of CB1 receptor signal in psychiatric disease like depression [18]. The interplay of cannabidiol and endocannabinoids [7] promote the activation of 5-HT1A receptors [50], so a possible connection of the endocannabinoid system and the serotonergic system in case of depression is supposable. There is evidence that increased endocannabinoid concentration and the stimulation of CB1 receptors reduce stress responses and anxiolytic effects [3, 21]. The fact that glucocorticoids modulate the excitability of 5-HT neurons results in an increased concentration of endocannabinoids. This gave an indication for a potential cellular mechanism, which is involved in the regulation of stress related behaviour [58]. Meanwhile, it is also known that the CB1 receptors are located on serotonergic neurons of the raphe nuclei. This implies that the endocannabinoid System modulates serotonergic functions [22]. These clinical results and our assumption indicate a relation between the serotonergic system and the endocannabinoid system in depression.

There are some limitations of the study. The small size of the genetic sample limits the power to detect small differences. Furthermore, the investigation comprised only one SNP of CNR1 and FAAH genes, there are many more applicable SNP within these genes. Due to the limited sample size, it was not possible to assess the differences that might exist between heterozygosity GA and homozygosity AA. Further limitation is the involvement of healthy volunteers. Extension of our conclusions to the major depression requires further studies on patients.

## Conclusion

In conclusion, our study shows no differences in serotonergic activity and plasma endocannabinoid concentration with respect to the genotype distribution of CNR1 and FAAH genes in healthy subjects. Finally, these results show that healthy subjects who are carriers of the CNR1-A allele have lower endocannabinoid concentrations than patients with major depression. Our results suggest that carriers of the CNR1-A allele may be more susceptible to the development of depression. In addition, an interaction between endocannabinoid concentration and the serotonergic system was demonstrated.

## Data Availability

Raw data were generated at LWL-University Hospital Bochum. Derived data supporting the findings of this study are available from the corresponding author [B.E.] on request.

## Abbreviations

µL: Microliter
2AG: 2-Arachidonylglycerol
5-HTT: Serotonin transporter
A: Adenosine
AEA: Anandamide
Ag: Silver
AgCl: Silver chloride
BDI: Beck Depression Inventory
BPRS: Brief Psychiatric Rating Scale
C: Cytosine
CB: Cannabinoid
CNR1: Cannabinoid receptor 1
Cz: Central zero
dB SPL: Decibel
eCS: Endocannabinoids
EDTA: Ethylendiaminotetraacetat
FAAH: Fatty acid amide hydrolase
FCz: Frontal central zero
G: Guanine
HAMD-21: Hamilton Depression Rating Scale
Hz: Hertz
kΩ: Kilo ohms
LDAEP: Loudness dependence of auditory evoked potentials
MAGL: Monoacylglycerol lipase
min: Minutes
ms: Milliseconds
MWT-B: Multiple Choice Word Test
N/P: Negativ/positiv
nM: Nanomol
PCR: Polymerase chain reaction
rpm: Rounds per minute
SERT: Serotonin receptor gene
SNP: Single-nucleotide polymorphism
STAI: State-Trait Anxiety Inventory
UPLC-MS/MS: Tandem mass spectrometer
